# Impaired fasting glucose and left ventricular diastolic dysfunction in middle-age adults: a retrospective cross-sectional analysis of 2971 subjects

**DOI:** 10.1186/s12933-015-0282-4

**Published:** 2015-09-15

**Authors:** Assi Milwidsky, Elad Maor, Shaye Kivity, Anat Berkovitch, Sagit Ben Zekry, Alexander Tenenbaum, Enrique Z. Fisman, Aharon Erez, Shlomo Segev, Yechezkel Sidi, Ilan Goldenberg, Rafael Kuperstein

**Affiliations:** Leviev Heart Center, Chaim Sheba Medical Center, Ramat Gan, Israel; Department of Internal Medicine C, Chaim Sheba Medical Center, Ramat Gan, Israel; Institute for Medical Screening, Chaim Sheba Medical Center, Ramat Gan, Israel; Pinchas Borenstein Talpiot Medical Leadership Program, Chaim Sheba Medical Center, Ramat Gan, Israel; Cardiac Rehabilitation Institute, Chaim Sheba Medical Center, Ramat Gan, Israel; Department of Internal Medicine “E”, Tel-Aviv Medical Center, 6 Weizmann Street, 64239 Tel Aviv, Israel; Sackler School of Medicine, Tel-Aviv University, Tel Aviv, Israel; Cardiovascular Diabetology Research Foundation, Holon, Israel

**Keywords:** Diastolic dysfunction, Diabetes mellitus, Impaired fasting glucose

## Abstract

**Background:**

Left ventricular (LV) diastolic dysfunction (LVDD) is a well-established and early echocardiographic characteristic of diabetic cardiomyopathy. However, there are limited data on the association between impaired fasting glucose (IFG) and LVDD.

**Objective:**

To determine whether IFG is associated with LVDD among middle age adults.

**Methods:**

Amongst 3781 subjects screened in an annual health survey program and referred for an echocardiogram, 2971 individuals without LV systolic dysfunction or valvular heart disease were selected. Mean age of study population was 59 ± 12 years and 75 % were men. The subjects were categorized into three groups: euglycemia (N = 2025), IFG (N = 534) and diabetes mellitus (DM; N = 412). Doppler echocardiography readers were blinded to glycemic state. Subjects with impaired LV relaxation, pseudo-normal or restrictive filling patterns were defined as having LVDD.

**Results:**

LVDD was diagnosed in 574 (19 %) of subjects and it was more prevalent among patients with IFG and DM than in euglycemic individuals (27, 30 and 15 %, respectively; p < 0.001). Patients with IFG and DM had lower ratios of early (E) to late (A) trans-mitral flow (0.9 ± 0.3 and 0.9 ± 0.3 vs. 1.1 ± 0.4, respectively, p < 0.001). LV hypertrophy (LVH) was also more prevalent among patients with IFG and DM (11 and 18 %, respectively, vs. 9 %; p < 0.001). Multivariate binary logistic regression model adjusted to age, gender, obesity, LVH, renal function, total, high and low density lipoprotein cholesterol, triglycerides, ischemic heart disease, hypertension and LV ejection fraction showed that patients with IFG were 43 % more likely to have LVDD compared with euglycemic subjects (95 % confidence interval 1.12–1.83, p = 0.004).

**Conclusions:**

IFG is independently associated with a significant increase in the likelihood for the presence of LVDD in middle aged adults.

## Background

Impaired fasting glucose (IFG) is a harbinger of diabetes mellitus (DM) [1], a major risk factor for cardiovascular morbidity and heart failure (HF) [2]. Early derangements in glucose metabolism are related to cardiovascular morbidity [3–5]. Previous studies have suggested that one of the earliest structural changes related to DM is left ventricular (LV) diastolic dysfunction (LVDD), preceding the emergence of systolic dysfunction and HF [6–9]. Early cardiac damage as reflected by cardiac chamber enlargement, left ventricular hypertrophy (LVH) and LV flow changes were shown to be related to impaired glucose tolerance (IGT) [10–12]. However a possible association between IFG and LVDD is controversial [13, 14]. Thus, the aim of the current study was to investigate whether IFG is associated with LVDD among a large group of middle age adults.

## Methods

### Study population

The study population comprised subjects who were referred for an echocardiographic examination as part of an annual medical screening program in a tertiary Medical Center. Study population was described in previous works [15–18], and includes mainly apparently healthy men and women who pass annual health screening survey examinations. Shortly, all participants are interviewed during each annual examination using standard questionnaires that gather data regarding demographic characteristics, health related habits (e.g. degree of physical activity) and medical history. An attendant physician at the center performs a complete medical interview and physical examination, including blood pressure measurement. Thereafter, blood samples are collected after a 12 h fast and analyzed immediately. All subjects undergo a maximal exercise stress test according to the Bruce protocol in each check-up. A computerized database of all annual visits in this center, since the year 2000 serves as the source of data for this study. The institutional review board of the Sheba medical center approved this study on the basis of strict maintenance of participants’ anonymity during database analysis, accordingly no individual consent was obtained.

From 01.02.2004 to 31.01.2014—a total of 22,402 subjects were screened in the annual program and 3871 of them were referred for echocardiography at the discretion of their attending physician. Subjects with systolic dysfunction (LVEF <50 %) or valvular diseases were excluded (N = 376). Additional 508 subjects were excluded due to presence of atrial fibrillation or time to completion of echocardiography of >1 year. Thus, the final study sample comprised 2971 subjects.

### Echocardiographic examination and parameters

Two-dimensional transthoracic echocardiographic and Doppler studies were obtained with clinical ultrasound machines equipped with 3.5 MHz transducers using standard views. Since 2004 the studies were digitally stored (McKesson’s Horizon Cardiology™ Medical Software, Tel-Aviv, Israel). LV systolic function was visually estimated by echocardiography specialists. LVDD was defined as such by experienced readers in the echocardiographic exam summary and was determined according to accepted guidelines at the time of the study performance [19, 20], through assessment of cardiac function by pulsed-wave Doppler examination of mitral inflow and Doppler tissue imaging of the mitral annulus. All subjects with impaired LV relaxation, pseudo-normal or restrictive filling patterns were defined as having LVDD.

Interventricular diastolic septal thickness (IVSd), LV diastolic diameter (LVDd), LV systolic diameter (LVDs) and left atrial diameter were determined. Peak velocities of early (E) and late (A) trans-mitral flow and deceleration time (DT) were determined, and the ratio E/A was calculated. LV mass (LVM) was determined according to the formula introduced by Devereux et al. [21] and normalized according to body surface area (BSA) to produce LV mass index (LVMI), adjustments of LVM to Ht^2.7^ were also made to correct for the effect of obesity on LVM evaluation [22]. LVH was determined separately by each correction (BSA and Ht^2.7^) according to the American society of cardiology and European association of echocardiography guidelines. Thus, LVH was defined as LVM/BSA >95 kg/m^2^ for women and >115 kg/m^2^ for men, and as LVM/Ht^2.7^ >44 g/m^2.7^ for women and >48 g/m^2.7^ for men, for each criteria separately [23].

We also calculated the relative wall thickness (RWT) (measured as: twice the posterior wall thickness divided by LVDd) and determined the LV anatomical pattern in each participant (i.e. normal LV, concentric LV remodeling, concentric LVH and eccentric LVH) [24]. Normal LVM and RWT were defined as normal LV anatomy, normal LVM and RWT >0.42 as concentric LV remodeling, increased LVM and RWT >0.42 as concentric LVH and increased LVM in the presence of RWT <0.32 as eccentric LVH.

### Definitions

IFG was defined according to current guidelines [25] as a 12 h fasting plasma glucose measurement between 100 and 125 in subjects without known DM. Blood samples were drawn before echocardiography (mean time to echocardiography: 74 ± 83 days). Diabetes mellitus was defined according to past or current diagnosis of DM or a fasting plasma glucose level >125 mg/dL or the use of hypoglycemic drugs. The remainder participants were defined as euglycemic.

Obesity was defined according to body mass index (BMI)—normal <25 kg/m^2^, overweight >25–30 kg/m^2^ and obese >30 kg/m^2^. Renal function was assessed using Cockroft–Gault formula to produce creatinine clearance (Crcl). Hypertension was defined as the presence of two blood pressure measurements >140/90 mmHg in two different occasions, a past medical diagnosis or treatment with anti-hypertensive drugs.

### Statistical analysis

Continuous data were compared with student t test and one-way ANOVA. Categorical data were compared with the use of Chi square test or Fischer exact test. Trend analysis was done using polynomial one-way-anova test for parametric and the Jonckheere–Terpstra test for non-parametric variables. Multivariate binary logistic regression modeling was used to evaluate the odds ratio of LVDD among subjects according to their glycemic state (euglycemic, IFG and DM). All models were further adjusted for the following specified covariates: age, gender, hypertension, obesity, LVH (determined by LVMI and LVM/Ht^2.7^), ejection fraction (EF), ischemic heart disease (IHD) and renal function. Odds ratios (and 95 % confidence intervals [CI]) for LVDD in subjects with DM and IFG were compared to euglycemic participants as the reference group.

In a confirmatory sub-analysis, we also divided the cohort into tertiles according to LVM and LV anatomical pattern in each participant (i.e. normal LV, concentric LV remodeling, concentric LVH and eccentric LVH). We then replaced the main definition of LVH (according to LVMI or LVM/Ht^2.7^) with these two definitions (i.e. LVM tertiles and the LV anatomic patterns) as covariates in two separate binary logistic regression models (with LVDD as the dependent variable).

Interaction-term analysis was used to evaluate the consistency of the association between IFG and LVDD in specified risk subsets categorized by age, gender, creatinine clearance, hypertension, BMI, low and high density lipoprotein cholesterol levels and LV anatomical features (LVH, LVM tertiles and 4 LV anatomical subgroups). In this analysis IFG subjects were compared with euglycemics as the reference group, while those with DM were excluded. Interactions were tested separately, adjusted for all the other relevant covariates.

Statistical significance was accepted for 2-sided p < 0.05. The statistical analyses were performed with IBM SPSS version 20.0 (Chicago, IL, USA).

## Results

Among 2971 study participants, mean age was 59 ± 12 and 75 % were men. There were 343 (12 %) active smokers and mean BMI was 26.8 ± 4 kg/m^2^. Hypertension was diagnosed in 1366 (46 %), mean systolic and diastolic blood pressures were 129 ± 18/79 ± 10 mmHg, respectively.

Among study subjects, 2025 (68 %) were euglycemic, 534 (18 %) had IFG, and 412 (14 %) had DM. The clinical and laboratory characteristics of study subjects by the three glycemic categories are presented in Table 1. The frequency of baseline clinical cardiovascular risk factors, including an older age, higher BMI, increased creatinine level, hypertension, and the presence of ischemic heart disease, was lowest in the euglycemic group and highest in the DM group (p < 0.001 both for the overall difference among the three groups and for trend, for all parameters). Total, low and high density lipoprotein cholesterol (LDL-c and HDL-c, respectively) and triglyceride levels were all significantly different between groups (p < 0.001, for all).

**Table 1:** Baseline clinical characteristics of study population

	Euglycemia (2025)	IFG (534)	DM (412)	P	P for trend*
Age, years	57 ± 12	62 ± 11	65 ± 10	<0.001	<0.001
Gender, male	1410 (70 %)	450 (84 %)	347 (84 %)	<0.001	<0.001
Days to echo^a^	74 ± 83	71 ± 81	74 ± 84	0.63	0.62
Active smokers	237 (12 %)	53 (10 %)	53 (13 %)	0.34	0.91
Physically active	1554 (77 %)	410 (77 %)	303 (74 %)	0.25	0.12
IHD	188 (9 %)	74 (14 %)	110 (27 %)	<0.001	<0.001
BMI	26.1 ± 4	27.6 ± 4	28.5 ± 4	<0.001	<0.001
Overweight	860 (43 %)	261 (49 %)	199 (47 %)	<0.001	<0.001
Obese	299 (15 %)	129 (24 %)	136 (33 %)	<0.001	<0.001
Systolic BP (mmHg)	127 ± 18	134 ± 18	135 ± 17	<0.001	<0.001
Diastolic BP (mmHg)	79 ± 10	81 ± 10	79 ± 10	<0.001	0.03
Hypertensive	769 (38 %)	293 (55 %)	304 (74 %)	<0.001	<0.001
Glucose (mg/dL)	88 ± 8	106 ± 6	131 ± 36	<0.001	<0.001
Hemoglobin (g/dL)	14.2 ± 1	14.5 ± 1	14.0 ± 1	<0.001	0.36
CrCl (mL/min)	84 ± 24	84 ± 26	85 ± 27	0.98	0.85
T.C. (mg/dL)	185 ± 33	180 ± 34	159 ± 35	<0.001	<0.001
LDL-C (mg/dL)	113 ± 26	110 ± 26	95 ± 26	<0.001	<0.001
HDL-C (mg/dL)	50 ± 13	47 ± 11	44 ± 11	<0.001	<0.001
TG (mg/dL)	113 ± 58	131 ± 68	144 ± 83	<0.001	<0.001

*BMI* body mass index, *BP* blood pressure, *CrCl* creatinine clearance, *DM* diabetes mellitus, *IFG* impaired fasting glucose, *HDL-C* high density lipoprotein cholesterol, *LDL-C* low density lipoprotein cholesterol, *Overweight* 25 < BMI ≤ 30, *Obese* BMI >30, *T.C.* total cholesterol, *TG* triglycerides

* Trend analysis was done using polynomial one-way-Annova test for parametric and the Jonckheere–Terpstra test for non-parametric variables

^a^Mean number of days from blood glucose measurement to echocardiography exam

### Echocardiographic indices and LVDD

LVDD was diagnosed in 574 (19 %) subjects, 473 (94.8 %) of them had grade 1 diastolic dysfunction (impaired relaxation, defined by the presence of an E/A ratio <0.8, a deceleration time >200 ms and E/E′ relation <8 in the presence of an enlarged left atrium), 23 patients a pseudo-normal pattern (4.6 %) and 3 a restrictive filling pattern (0.6 %). The mean LVEF of the entire population was 60 ± 3 %.

Echocardiographic indices of study subjects by the three glycemic groups are presented in Table 2. LV diastolic dimension and IVSd were directly correlated with the level of dysglycemia (p < 0.001 for trend, for both). LVM, LVMI, LVM/Ht^2.7^ and left atrium area all significantly increased from normal to IFG and DM (p < 0.001 for the overall difference among the 3 groups and for trend for all comparisons). LVH (as adjusted either for BSA or Ht^2.7^) was significantly more prevalent in subjects with IFG and DM compared to those who were euglycemic (p < 0.001 for both adjustments). E/A ratios were lower in subjects with IFG and DM as compared to euglycemics (p < 0.001). There was a non-significant increase in deceleration time among the 3 groups. E/E′ (early mitral flow to early mitral annular movement) ratio significantly increased with the level of dysglycemia (p < 0.001 both for the overall difference among the three groups and for trend).

**Table 2:**
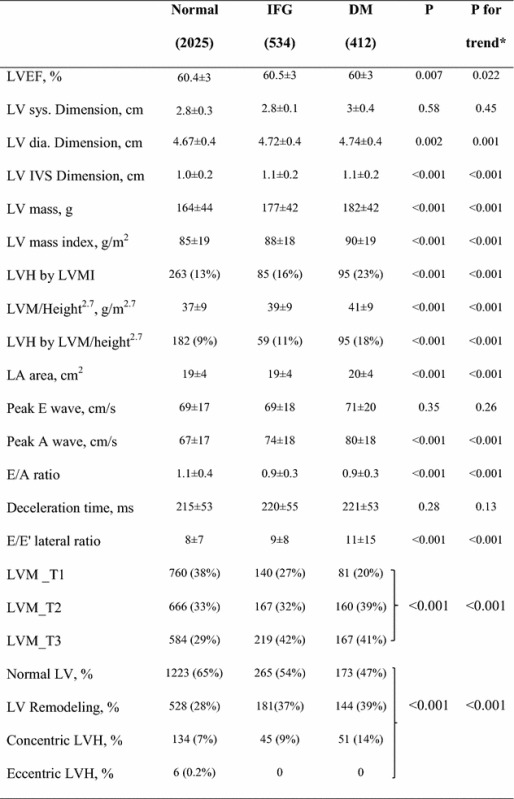
Echocardiographic parameters of study groups

*BSA* body surface area, *E’* mitral annular movement as measures by tissue Doppler, *EF* ejection fraction, *Dia.* diastole, *IVS* inter ventricular septum, *LA* left atrium, *LV* left ventricle, *LVDD* left ventricular diastolic dysfunction, *LVM_T* LV mass tertile, *Ms* milliseconds, *RWT* relative wall thickness, *Sys.* systole

* Trend analysis was done using polynomial one-way-Annova test for parametric and the Jonckheere–Terpstra test for non-parametric variables

Notably, of 574 participants diagnosed with LVDD, only a minority met the criteria for LVH—159 (28 %) according to LVMI and 117 (20 %) according to LVM/Ht^2.7^.

### LVDD and glycemic groups

**Fig. 1:**
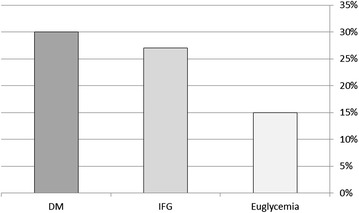
Rate of LVDD among subjects in different glycemic groups [308 (15 %) of euglycemic, 142 (27 %) of those with IFG and 124 (30 %) of diabetic participants]. *p < 0.001 for the comparison between both IFG and DM to euglycemic groups

The prevalence of LVDD was significantly higher among subjects with both IFG (27 %) and DM (30 %), as compared to euglycemic participants (15 %) (p < 0.001 for the overall difference among the three groups, and for the comparison between both the IFG and DM groups with euglycemic group; Fig. 1).

Univariate binary logistic regression model showed that subjects with IFG and DM were significantly more likely to have LVDD (OR = 2.01 95 % CI [1.61–2.35] and OR = 2.40, 95 % CI [1.88–3.06], respectively; p < 0.001 for both comparisons). Multivariate binary logistic regression analysis showed consistent findings (Table 3) After adjustment for age, gender, obesity, creatinine clearance, hypertension, IHD, EF, smoking status, total cholesterol, triglycerides level, LDL-c, HDL-c and LVH (determined by either LVMI or LVM/Ht^2.7^)—IFG and DM were shown to be independently associated with a 43 and 38 % increased likelihood for the presence of LVDD, respectively (p = 0.004 for IFG and p = 0.03 for DM).

**Table 3:** Binary logistic regression: effect of glycemic group on the risk for left ventricular diastolic dysfunction

	OR	95 % CI	P value
IFG vs. normal	1.43	1.12–1.83	0.004
DM vs. normal	1.38	1.04–1.83	0.03
Age (for each 1 year)	1.07	1.06–1.09	<0.001
Gender, male	1.15	0.88–1.52	0.30
Hypertension	1.33	1.07–1.66	0.01
LVH	2.05	1.60–2.62	<0.001
BMI >25	1.44	1.12–1.84	0.005

Model is further adjusted to LVEF, IHD, renal function, smoking status, TC, TG, LDL-C and HDL-C

*CI* confidence interval, *DM* diabetes mellitus, *HDL-c* high density lipoprotein cholesterol, *IFG* impaired fasting glucose, *IHD* ischemic heart disease, *LDL-c* low density lipoprotein cholesterol, *LVEF* left ventricular ejection fraction, *LVH* left ventricular hypertrophy determined by left ventricular mass index (LVH/BSA), *OR* odds ratio, *TC* total cholesterol, *TG* triglycerides

Consistent findings were shown when the main definition of LVH was replaced by LVM tertiles or by the presence of an abnormal LV anatomical pattern (i.e. concentric LV remodeling, concentric LVH and eccentric LVH) as covariates in the multivariate model. This analysis showed that IFG remained independently associated to LVDD when the main definition of LVH was replaced by LVM tertiles (OR = 1.40 95 % CI [1.09–1.80]; p = 0.009) or by LV anatomical pattern (OR = 1.40 95 % CI [1.09–1.80]; p = 0.008).

**Fig. 2:**
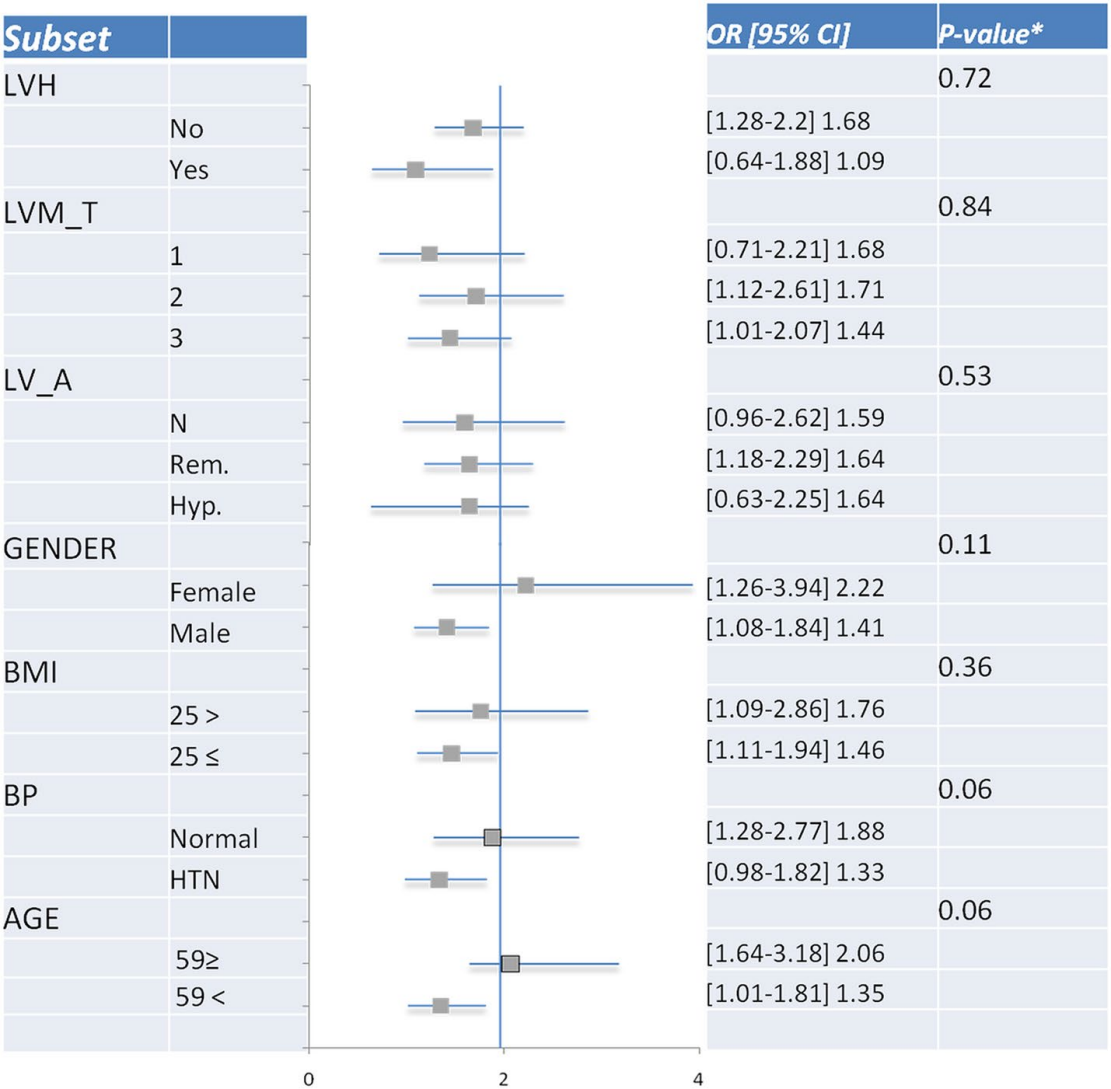
Odds ratios (and 95 % confidence intervals [CI]) for LVDD in IFG as compared with euglycemic subjects and, P values for interaction between IFG to specified (e.g. age and hypertension). The *vertical line* is the reference HR for LVDD in IFG compared to euglycemic subjects (=1.96). *LV_Anatomy* anatomical pattern, *LVM_T* LVM tertiles

Subgroup analysis was carried out in 10 specified risk subsets and is presented in Fig. 2. This analysis showed that the independent association between IFG and the likelihood for the presence of LVDD was consistent in subjects categorized by age, gender, Crcl, BMI, HDL-c, LDL-c, LVH, LVM tertiles and LV anatomy. No interaction between IFG to the specified variables was present. However, the association between IFG and LVDD was more pronounced among lower risk subjects, including those without a history of hypertension (adjusted OR = 1.88 95 % CI [1.28–2.77]) as compared to those with hypertension (adjusted OR = 1.33 95 % CI [0.98–1.82]; p value for IFG-by-hypertension interaction = 0.06), and among younger subjects (age <59; adjusted OR = 2.06 95 % CI [1.64–3.18]) as compared with older subjects (adjusted OR = 1.35 95 % CI [1.01–1.81]; p value for IFG-by-age interaction = 0.06).

## Discussion

The main finding of the current study is that IFG is independently associated with a significant increase in the likelihood for the presence of diastolic dysfunction in middle aged adults.

This finding is further substantiated by evidence of increased LV diastolic dimensions, increased LVM, LVMI, increased LVM/Ht^2.7^ and increased LA dimensions, among subjects with IFG. Notably, we have also shown that the association between IFG and LVDD was independent of LV anatomical abnormalities such as concentric LV remodeling and LVH, but appeared to be more pronounced among normotensive and younger subjects in whom LVDD is generally less prevalent.

### Prevalence of LVDD

The reported prevalence of diastolic dysfunction in the general population ranges from 11.1 to 34.7 % and is influenced by a number of factors including the characteristics of the population, the choice of imaging modalities and the criteria applied for diagnosing diastolic dysfunction [26]. In the present study, 574 (19 %) patients met the criteria for LVDD.

### DM and LVDD

The association between DM and cardiovascular morbidity is well documented, both through increased risk for the development of coronary artery disease (CAD) and to heart failure unassociated to the presence of CAD [2]. Diastolic dysfunction precedes progression to overt heart failure either with depressed or preserved EF in many diabetic subjects [9, 27]. Actually, a recent major prospective study in patients with long standing type 1 DM, found a prevalence of 3.7 % of CHF at the end of a 7 years follow up. Diastolic HF constituted 85 % of the cases of HF [28].

### IFG and LVDD

Early derangements in glucose metabolism are related to cardiovascular morbidity [3–5]. Elmm et al. followed 10,498 patients for a period of 5.2 years. Sixty five percent of the 298 patients who died during follow-up had a glucose metabolic disturbance at baseline. Importantly, IFG was associated with a hazard ratio of 2.5 for cardiovascular mortality [29].

Data regarding the association of IFG to LVDD is equivocal, for example while Shimabukuro et al. showed that IGT but not IFG is related to LVDD, Capaldo et al. recently found that both are related to reduced ratios of peak velocities of early (E) to late (A) trans-mitral flow and to increased LVM [13, 14].

Our work shows, in a large cohort of apparently healthy middle age adults that IFG is independently associated with LVDD. These results stress the fact that IFG might serve as a marker for a possible early cardiac involvement in the dysglycemic process even in the absence of other co-morbidities such as hypertension. It should be noted that both the European society for diabetes and the American diabetes association do not currently recommend routine echocardiographic screening of patients with diabetes or IFG [25, 30].

However, subclinical LVDD is recognized as an important predictor of heart failure and long-term mortality [31]. In contrast to diabetic societies current heart failure guidelines [32, 33] give special emphasis to the early detection of these asymptomatic changes of left ventricle function and the identification of its main risk factors.

In the present analysis prevalence of LVDD was similar in IFG and DM groups. This apparent paradox may be explained by the fact that many patients with DM who already suffered cardiac complications (like systolic HF, valvular disease and atrial fibrillation) were excluded from the analysis and those who left might represent a relatively healthier subgroup.

Even after multivariate analysis that included obesity, hypertension and LVH, IFG remained closely and independently related to LVDD. Interaction analysis showed that association between IFG and LVDD was strongest among young normotensive subjects (data presented in Fig. 2). This finding reinforces our hypothesis that dysglycemia itself may be related to diastolic dysfunction independently of other co-morbidities and anatomical abnormalities.

### Pathogenesis of diastolic dysfunction in dysglycemia and possible treatment directions

The mechanisms underlying the relation between dysglycemia and diastolic dysfunction are many, some grouped under the term “Diabetic Cardiomyopathy” claimed by Rubler et al. [34]. and currently under rigorous investigation. These mechanisms include—fibrosis and accumulation of advanced glycosylation end products in the myocardium, cardiomyocytes lipotoxicity and direct effects of insulin on the myocardium and its vasculature, including cellular apoptosis, endothelial dysfunction and chronic adrenergic stimulation [6, 35]. Other important mechanisms included presence of insulin resistance [36–38], excessive visceral adipose tissue [39, 40], activity of circulating dipeptidyl peptidase-4 [41, 42] and fatty acid-binding protein-4 [43].

Lifestyle intervention based on dietary management and physical activity is a well-established approach to the management of various cardiometabolic diseases, including diabetes, obesity and metabolic syndrome. Increasing evidence reports favorable and specific effects of lifestyle interventions on central obesity, insulin-resistance, glucose intolerance and myocardial function [44–46].

## Limitations

The main strength of our study is the relatively large cohort size under rigorous follow-up. Despite of that there are several limitations: First, this is a cross-sectional retrospective study of a selected group with risk of residual confounding. This is reinforced by the nature of our population, with a low prevalence of obesity, a good physical condition (e.g. 76 % were physically active) and an early and effective treatment for their hypertension (e.g. reflected by adequate mean blood pressure values, see Table 1). Second, the relatively high prevalence of hypertension in our study population probably could be a result from a referral bias because hypertensive patients are commonly referred for echocardiography as part of our screening program. Finally, left ventricular function was visually estimated, reflecting common clinical practice [47, 48].

## Conclusion and implications

The systematic evaluation of diastolic function by echocardiography as established by the current guidelines enables a more accurate identification of cardiac abnormalities in at risk patients at an earlier and hopefully reversible stage of their disease. Our work shows that IFG is associated to LVDD among middle age adults independently of numerous factors, including concentric remodeling, LVH and obesity. These findings support evaluation of diastolic function in subjects with IFG for possible early cardiac involvement in dysglycemic process. Future prospective longitudinal studies are required to assess long-term prognosis and the reversibility of LVDD through life style modification, drug treatment and other medical interventions in these patients.

## Abbreviations

BMI: body mass index
BP: blood pressure
CrCl: creatinine clearance, as estimated by the Cockroft–Gault formula
DM: diabetes mellitus
E/A ratio: ratio of early (E) to late (A) trans-mitral flow
EF: ejection fraction
HDL-c: high density lipoprotein cholesterol
HF: heart failure
Ht: height
HTN: hypertension
IFG: impaired fasting glucose
IHD: ischemic heart disease
LDL-c: low density lipoprotein cholesterol
LV: left ventricle
LVDD: left ventricular diastolic dysfunction
LVEF: left ventricular ejection fraction
LVH: left ventricular hypertrophy
LVM: left ventricle mass
